# Benefits of Cartilage Conduction Hearing Aids for Speech Perception in Unilateral Aural Atresia

**DOI:** 10.3390/audiolres11020026

**Published:** 2021-06-17

**Authors:** Sakie Akasaka, Tadashi Nishimura, Hiroshi Hosoi, Osamu Saito, Ryota Shimokura, Chihiro Morimoto, Tadashi Kitahara

**Affiliations:** 1 Department of Otolaryngology-Head and Neck Surgery, Nara Medical University, 840 Shijo-cho, Kashihara, Nara 634-8522, Japan; sakasaka@kcn.jp (S.A.); o-saito@naramed-u.ac.jp (O.S.); mori-chi@naramed-u.ac.jp (C.M.); tkitahara@naramed-u.ac.jp (T.K.); 2 MBT (Medicine-Based Town) Institute, Nara Medical University, 840 Shijo-cho, Kashihara, Nara 634-8522, Japan; hosoi@naramed-u.ac.jp; 3 Graduate School of Engineering Science, Osaka University, D436, 1-3 Machikaneyama, Toyonaka, Osaka 560-8531, Japan; rshimo@sys.es.osaka-u.ac.jp

**Keywords:** atretic ear, unilateral conductive hearing loss, bone conduction, diotic summation, speech recognition

## Abstract

Severe conductive hearing loss due to unilateral aural atresia leads to auditory and developmental disorders, such as difficulty in hearing in challenging situations. Bone conduction devices compensate for the disability but unfortunately have several disadvantages. The aim of this study was to evaluate the benefits of cartilage conduction (CC) hearing aids for speech perception in unilateral aural atresia. Eleven patients with unilateral aural atresia were included. Each participant used a CC hearing aid in the atretic ear. Speech recognition scores in the binaural hearing condition were obtained at low speech levels to evaluate the contribution of aided atretic ears to speech perception. Speech recognition scores were also obtained with and without presentation of noise. These assessments were compared between the unaided and aided atretic ear conditions. Speech recognition scores at low speech levels were significantly improved under the aided atretic ear condition (*p* < 0.05). A CC hearing aid in the unilateral atretic ear did not significantly improve the speech recognition score in a symmetrical noise presentation condition. The binaural hearing benefits of CC hearing aids in unilateral aural atresia were predominantly considered a diotic summation. Other benefits of binaural hearing remain to be investigated.

## 1. Introduction

Unilateral hearing deficit deprives individuals of the benefits of binaural hearing naturally present in individuals with normal hearing and disturbs auditory development [1,2,3,4]. Thus, auditory intervention is required for unilateral hearing disability as well as for binaural disability. Representative benefits of binaural hearing are diotic summation, binaural squelch, and improved sound localization [5].

Air conduction (AC) hearing aids are usually used as an intervention device in most individuals with hearing loss. However, some pathological ear conditions, such as atretic ear, prevent the use of AC hearing aids. Bone conduction (BC) hearing aids are effective in atretic ears and are therefore used instead of AC hearing aids in individuals with aural atresia. Unfortunately, BC hearing aids also have several disadvantages concerning comfort, esthetics, and stability [5,6]. Its alternatives include implantable BC devices [7,8,9,10], which unfortunately require surgical intervention. For most patients with unilateral aural atresia, these options are not desired.

On attaching a transducer on the aural cartilage, the patient is able to perceive loud sounds [11], and this conduction, termed cartilage conduction (CC), has characteristics different from those of conventional AC and BC [12,13,14,15,16]. CC hearing aids are new, innovative hearing devices utilizing CC, which address the issues concerning the fixation of BC hearing aids and require no surgical intervention [17,18,19,20,21]. Thus, they can be an attractive alternative for patients with unilateral aural atresia.

Hearing via CC is not simple, since both direct-AC sound and airborne sound generated by vibrating the cartilaginous portion of the ear canal result in sound perception [22]. CC hearing provides excessive low-frequency boost depending on the ear conditions [23], which can deteriorate speech perception [24]. However, appropriate gain-adjustment can improve it [25], and according to the previous reports on CC hearing aids, patients with aural atresia had good speech recognition in the aided condition [20]. In Japan, CC hearing aids have been clinically used since 2017 and gained popularity among patients with aural atresia [26,27,28]. A nationwide clinical survey revealed excellent outcomes of CC hearing aids in the patients who experienced difficulty with AC hearing aids due to aural atresia, canal stenosis, and chronic continuous otorrhea [29].

In clinical use, patients who tried CC hearing aids reported benefits, such as improved conversation in noisy situations and improved sound localization, and they wished to continue using them [20]. Our previous study revealed improved sound localization with CC hearing aid use in patients with bilateral aural atresia [30]. In contrast, the benefits of CC hearing aid in unilateral aural atresia remain unclear. Unilateral aural atresia causes unilateral severe conductive hearing loss, since the patient is deprived of unilateral AC due to a lack of the ear canal. Amplification in the ear affected by unilateral severe hearing loss with a hearing aid improves binaural hearing, which contributes to improved speech recognition, conversation in noisy situations, and sound localization [31,32]. It remains to be investigated whether these binaural hearing benefits are provided with a CC hearing aid in the unilateral atretic ear. The purpose of this study was to clarify the audiological benefits of CC hearing aids for the unilateral atretic ear. The contributions of CC hearing aid to speech perception by the unilateral atretic ear were investigated.

## 2. Materials and Methods

All participants were recruited from a previous clinical trial of CC hearing aids [20]. Eleven participants (three females; eight males) with unilateral aural atresia who used CC hearing aids were enrolled in the present study. The median age of the participants was 29 years (range, 7–83 years). The average AC and BC hearing levels at 500, 1000, and 2000 Hz in pure tone audiometry of atretic ears were 68.9 ± 15.9 dB and 17.7 ± 8.7 dB, respectively. The average AC for unaffected ears was 14.7 ± 10.8 dB. The study was approved by the ethics committee of Nara Medical University (No. 09-KEN011). Participants provided written informed consent before being enrolled. If the participant’s age was <20 years, the parents provided consent.

The average threshold at 500, 1000, and 2000 Hz in atretic ears aided with a CC hearing aid was 35.6 ± 9.0 dB. When the thresholds in the atretic ear were measured in the sound field, normal ear was masked with narrow band noise. In some participants, adequate masker level could not be determined using a plateau method due to a large difference between the two ears, and the unaided threshold in the atretic ear (and functional gain) could not be obtained. Judging from the aided threshold and functional gain in bilateral atretic ears in the previous study, the functional gains for the participants were estimated to be 30–40 dB [20]. The duration of CC hearing aid use was 36.8 ± 11.2 months, while nobody had used other hearing devices before the fitting. The CC hearing aids used in this study were equipped with the directional mode and noise suppression functions. However, these functions had not been activated both for daily use and during the measurement in all subjects.

### 2.1. Measurement of Speech Recognition at Low Speech Levels

**Figure 1:**
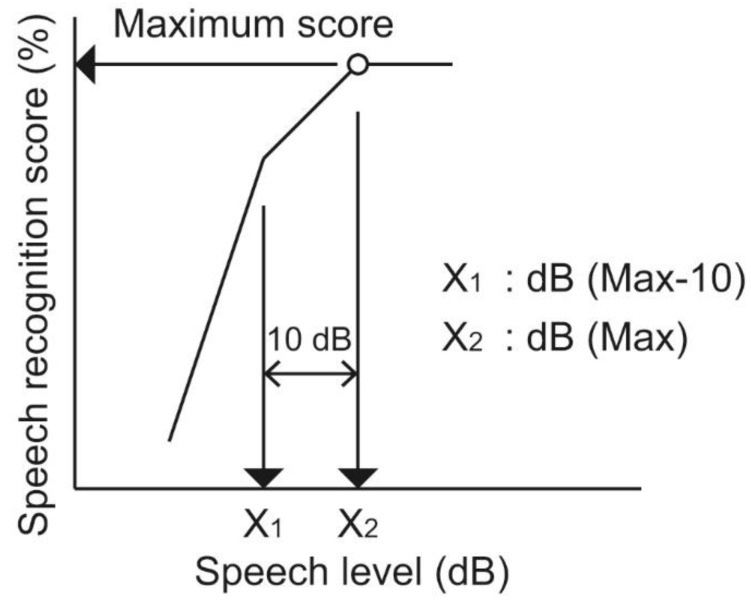
Determination of the dB (Max) and dB (Max-10). Speech performance-intensity function under the unaided binaural hearing condition was measured using 20-monosyllable word lists in 10-dB steps. The minimum speech level at which the maximum speech recognition score was obtained was termed the “dB (Max)” (X_2_ in the figure); the “dB (Max-10)” was determined by subtracting 10 dB from the dB (Max) (X_1_ in the figure).

The contribution of CC hearing aids in atretic ears to speech recognition was estimated. The normal ears allowed conversation in quiet environments. The contribution of CC hearing aids in quiet situations is difficult to estimate at more than a moderate speech level. Speech recognition scores were obtained at low speech levels under the unaided and aided conditions, and the scores were compared. In Japan, speech audiometry is conducted using 57-S or 67-S word lists including 50- or 20-monosyllable words, respectively. They are authorized by the Japan Audiological Society [33]. In order to evaluate speech recognition in detail, 57-S word lists are preferable as the test material owing to the larger number of the monosyllables. However, a long examination time is required for the repeated measurements using 57-S word lists. To reduce the burden of the examination, speech performance-intensity functions were first measured using 67-S word lists. Speech recognition was measured in 10-dB steps under the unaided condition. The speech level at which the maximum score was obtained in the speech performance-intensity function was defined as the “dB (Max)”. After the dB (Max) was determined using 67-S word lists for each participant, speech recognition tests using the 57-S word lists were conducted under the unaided and aided conditions. The measurements were conducted not only at the dB (Max), but also at 10 dB below the dB (Max), which was defined as the “dB (Max-10).” In this study, dB (Max-10) was employed as a low speech level. The determination procedures of the dB (Max) and dB (Max-10) are described in Figure 1.

### 2.2. Measurement of Speech Recognition in Noise

The speech recognition scores with and without noise were compared under the unaided and aided conditions. A loudspeaker for speech presentation was located 1 m in front of each participant. Two loudspeakers for noise presentation were individually located at ±45 degrees azimuth at a distance of 1 m according to ISO 8253-3 (2012). The 57-S word lists and speech-weighted noise were employed as the test material and noise, respectively. The power spectrum of the speech-weighted noise was constant from 125 Hz to 1000 Hz, with a roll-off of 12 dB/oct [34]. The presented noise between two loudspeakers was uncorrelated. Speech recognition scores were obtained at a 60-dB hearing level in the unaided and aided binaural hearing conditions, and the measurements were performed with and without noise presentation. The signal-to-noise ratio (SNR) was set at +10 dB. These procedures were performed according to guidelines that are standard in Japan [35].

The above-mentioned assessments were performed in a soundproof room (dimensions, approximately 5.4 m × 5.4 m). The calibration of the loudspeakers was carried out with a sound level meter (NA–20; Rion, Kokubunji, Japan).

### 2.3. Statistical Analysis

Speech recognition scores at two speech levels were analyzed using two-way analysis of variance (ANOVA), with hearing aid (aided with CC hearing aid or not) and speech levels as within-subject factors. The impact of noise on speech recognition scores were also analyzed using two-way ANOVA, with hearing aid and noise (with and without noise presentation) as within-subject factors. Statistical ANOVA was performed using SPSS ver. 22 (International Business Machines Corporation, Armonk, NY, USA). The Bonferroni method was used as a post-hoc correction of the multiple comparisons test after ANOVA. Significance was set at 0.05.

## 3. Results

**Figure 2:**
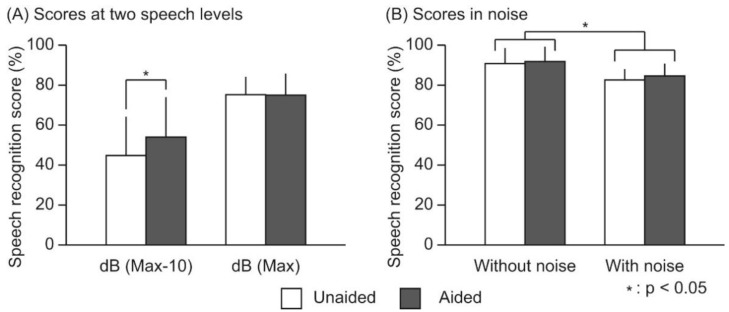
Speech recognition scores at two speech levels (**A**) and with/without noise presentation (**B**). Speech recognition scores were measured using 50-monosyllable word lists in unaided and aided binaural hearing conditions. Vertical bars indicate standard deviations.

The obtained speech performance-intensity functions determined the dB (Max) of each participant. The average dB (Max) was 35.4 ± 12.1 dB. Figure 2A shows the speech recognition scores at the dB (Max) and dB (Max-10) under the unaided and aided conditions. ANOVA revealed a significant effect for speech level (F(10, 1) = 37.57, *p* < 0.01), but not for the hearing aid (F(10, 1) = 3.07, *p* = 0.11). A significant interaction between them was found (F(10, 1) = 7.54, *p* < 0.05). In the post-hoc tests at the dB (Max-10), the speech recognition score under the aided condition was found to be 54.0 ± 20.0%, which was significantly higher than that under the unaided condition, which was 44.7 ± 19.4% (*p* < 0.05).

The speech recognition scores decreased under the noise presentation condition (Figure 2B). ANOVA revealed a significant effect for noise (F(10, 1) = 12.20, *p* < 0.01), but not for the hearing aid (F(10, 1) = 1.56, *p* = 0.24). No interaction between them was found (F(10, 1) = 0.14, *p* = 0.72). Speech recognition scores significantly decreased with noise presentation. No differences in the decrease were found between the unaided and aided conditions.

## 4. Discussion

The benefits of CC hearing aids in unilateral aural atresia were evaluated. We investigated the effects of a CC hearing aid in the atretic ear on speech recognition at low speech levels and in presence of noise. Only the benefit of diotic summation on speech recognition was obtained in this study.

Unilateral aural atresia induces severe conductive hearing loss of the atretic ear, which causes a large difference between the two ears. The amplification gain with CC hearing aids is estimated to be 30–40 dB [20], reducing the left–right difference in hearing. This study tried to evaluate the contribution of CC hearing aids in atretic ears to speech recognition. When the presentation level is high enough for the normal ear alone to accurately understand the speech, the contribution of the atretic ear cannot be detected. Therefore, the speech recognition scores under the unaided and aided conditions were obtained at 2 presentation levels: dB (Max) and dB (Max-10). Although no difference in speech recognition scores was observed at the dB (Max), binaural hearing benefit on speech recognition was observed at the dB (max-10). Low speech level condition revealed the contribution of the aided atretic ear to speech recognition. Diotic summation contributes to improving speech recognition in difficult hearing conditions [36]. The sound condition in daily conversation is poorer than that in the experimental room. CC hearing aids in atretic ears are expected to assist real-life listening by the diotic summation.

Another advantage of binaural hearing is improved hearing in noisy situations. Individuals with binaural hearing can benefit from head shadow effects just by attending to the ear with the better SNR [37]. Furthermore, the auditory system can combine different mixtures of speech and noise arriving at each ear to effectively remove some of the noise [38]. Unfortunately, no binaural hearing benefit was identified in this study. The reduction of speech intelligibility due to noise has been associated with various factors, including localization of noise, SNR, and type of noise [39,40]. Speech-weighted noises were presented from ±45 degrees azimuth according to ISO 8253-3 (2012), which symmetrically disturbed the hearing in both ears. In such a noise presentation condition, binaural squelch did not function well. In previous studies, the speech recognition under noise condition was improved with a bone anchored hearing aid (BAHA) in the atretic side [41,42]. However, those evaluations were conducted with different arrangements of signal and noise presentation. They placed the loudspeakers for noise presentation contralateral to the BAHA side. In this noise presentation condition, binaural squelch provided its benefits. If the current measurements were conducted in similar noise presentation condition as these previous studies, the benefits would be observed.

### Limitations of the Study

Most patients with unilateral aural atresia who have tried CC hearing aids in atretic ears wished to continuously use the aids, as they subjectively perceived the benefits of binaural hearing after daily use [20]. This study evaluated the benefits of binaural hearing in terms of speech recognition and speech recognition under noise presentation. However, significant improvement was objectively observed only for speech recognition at low speech levels. The other factors such as age and laterality probably influence the benefits of binaural hearing. The sample size of this study was too small to determine the impact of these factors. In terms of the experimental condition, the arrangement of loudspeakers and the type of noise present have to be reconsidered. Further study is required to elucidate the benefits of CC hearing aids in a unilateral atretic ear.

## 5. Conclusions

The benefits of binaural hearing with CC hearing aids in unilateral aural atresia were evaluated. By decreasing the left–right difference in hearing, speech recognition scores improved at low speech levels. No improvements in speech recognition in noise were found. The binaural hearing benefits of CC hearing aids in unilateral aural atresia were predominantly considered to be a diotic summation.

## Data Availability

Not applicable.
